# Active repression by Blimp1 play an important role in osteoclast differentiation

**DOI:** 10.1186/ar3655

**Published:** 2012-02-09

**Authors:** Keizo Nishikawa, Tomoki Nakashima, Mikihito Hayashi, Takanobu Fukunaga, Shigeaki Kato, Tatsuhiko Kodama, Satoru Takahashi, Kathryn Calame, Hiroshi Takayanagi

**Affiliations:** 1 Laboratory of Cellular Dynamics Immunology Frontier Research Center, Osaka University, Yamada-oka 3-1, Suita, Osaka 565-0871, Japan; 2 Department of Cell Signaling, Graduate School, Tokyo Medical and Dental University, Yushima 1-5-45, Bunkyo-ku, Tokyo 113-8549, Japan; 3 Global Center of Excellence Program, International Research Center for Molecular Science in Tooth and Bone Diseases, Japan; 4 Japan Science and Technology Agency, ERATO, TakayanagiOsteonetwork Project, Yushima 1-5-45, Bunkyo-ku, Tokyo 113-8549, Japan; 5 Institute of Molecular and Cellular Biosciences, Graduate School of Medicine, University of Tokyo, Tokyo 113-0032, Japan; 6 Japan Science and Technology Agency, ERATO, Kato Nuclear Complex, Saitama 332-0012, Japan; 7 Department of Molecular Biology and Medicine, Research Center for Advanced Science and Technology, University of Tokyo, Komaba 4-6-1, Meguro-ku, Tokyo 153-8904, Japan; 8 Institute of Basic Medical Sciences and Laboratory Animal Resource Center, University of Tsukuba, Tennodai 1-1-1, Tsukuba 305-8575, Japan; 9 Department of Biochemistry and Molecular Biophysics, Columbia University College of Physicians and Surgeons, New York, NY 10032, USA

## 

Regulation of irreversible cell lineage commitment depends on a delicate balance between positive and negative regulators, which comprise a sophisticated network of transcription factors. Receptor activator of nuclear factor-κB ligand (RANKL) stimulates the differentiation of bone-resorbing osteoclasts through the induction of nuclear factor of activated T-cells c1 (NFATc1), the essential transcription factor for osteoclastogenesis. Osteoclast-specific robust induction of NFATc1 is achieved through an autoamplification mechanism, in which NFATc1 is constantly activated by calcium signaling while the negative regulators of NFATc1 are being suppressed. However, it has been unclear how such negative regulators are repressed during osteoclastogenesis. Here we show that B lymphocyte-induced maturation protein-1 (Blimp1; encoded by *Prdm1*), which is induced by RANKL through NFATc1 during osteoclastogenesis, functions as a transcriptional repressor of anti-osteoclastogenic genes such as *Irf8 *and *Mafb*. Overexpression of Blimp1 leads to an increase in osteoclast formation and *Prdm1*-deficient osteoclast precursor cells do not undergo osteoclast differentiation efficiently. The importance of Blimp1 in bone homeostasis is underscored by the observation that mice with an osteoclast-specific deficiency in the *Prdm1 *gene exhibit a high bone mass phenotype owing to a decreased number of osteoclasts. Thus, NFATc1 choreographs the cell fate determination of the osteoclast lineage by inducing the repression of negative regulators as well as its effect on positive regulators.

